# Open Study of the Safety and Efficacy of a Single Oral Dose of Cefixime for the Treatment of Gonorrhea in Pregnancy

**DOI:** 10.1155/S1064744997000434

**Published:** 1997

**Authors:** Joseph M. Miller

**Affiliations:** Department of Obstetrics and Gynecology Louisiana State University Medical Center 1542 Tulane Avenue New Orleans LA USA

## Abstract

*Objective:* The intent of this study was to determine the efficacy and tolerance of single dose oral cefixime use in the treatment of pregnant women with endocervical gonococcal carriage.

*Methods:* A retrospective review of clinic records over a 3 year period identified patients treated with a single 400 mg dose of cefixime for gonorrhea during pregnancy. Side effects and subsequent gonococcal carriage were noted.

*Results:* Pregnant women (n = 102) treated with cefixime were reviewed. A cure rate of 95.2% was found. Side effects were reported in three patients: two had nausea and vomiting and one had diarrhea.

*Conclusions:* A single 400 mg oral dose of cefixime was effective for the treatment of gonorrhea and was well tolerated by the pregnant women.


Infectious Diseases in Obstetrics and Gynecology 5:259–261 (1997)
© 1997 Wiley-Liss, Inc.

### Open Study of the Safety and Efficacy of a Single Oral Dose of Cefixime for the Treatment of Gonorrhea in Pregnancy

**Joseph M. Miller, Jr.***
*Department of Obstetrics and Gynecology, Louisiana State University Medical Center, New Orleans, LA*

ABSTRACT

*Objective:* The intent of this study was to determine the efficacy and tolerance of single dose oral cefixime use in the treatment of pregnant women with endocervical gonococcal carriage.

*Methods:* A retrospective review of clinic records over a 3 year period identified patients treated with a single 400 mg dose of cefixime for gonorrhea during pregnancy. Side effects and subsequent gonococcal carriage were noted.

*Results:* Pregnant women (n = 102) treated with cefixime were reviewed. A cure rate of 95.2% was found. Side effects were reported in three patients: two had nausea and vomiting and one had diarrhea.

*Conclusions:* A single 400 mg oral dose of cefixime was effective for the treatment of gonorrhea and was well tolerated by the pregnant women. Infect. Dis. Obstet. Gynecol. 5:259–261, 1997.
© 1997 Wiley-Liss, Inc.

KEY WORDS
STD screening; pregnancy; cefixime

Gonococcal carriage in pregnancy raises concern for the patient, her unborn child, and her sexual partner. Current treatment recommendations include intramuscular ceftriaxone and single dose oral treatment with cefixime, ciprofloxin, or ofloxacin.[1,2] Of oral medications, only cefixime is indicated for pregnant women.[2] When considering treatment in pregnancy, the oral cefixime would be both simpler to administer and painless. Cefixime appears highly effective.[1] We have used cefixime since July 1993, because alternative drugs for use in pregnancy are not freely available. We present our experience with cefixime concerning efficacy and tolerance in 102 patients followed in our practice. We are unaware of prior reports concerning cefixime use in pregnancy.

### SUBJECTS AND METHODS

The reported patients attended a community-based prenatal and family planning program in an underserved area between July 1993 and October 1996. Surveillance data previously identified gonococcal cervical carriage in just over 10% of prenatal patients.[3] Patients included in this report were identified by audit of clinic records.

Cervical swabs were analyzed by direct DNA assay (Gen-Probe, Inc., San Diego, CA), which was routinely performed on all obstetric registrants and repeated at 34 weeks in the author's clinic. This test has a sensitivity of 93–99% and a specificity of 99%.[4,5] Pregnant women with a positive gonorrheal screen were offered treatment with 400 mg of cefixime on site and observed for 30 min after this

*Correspondence to: Dr. Joseph M. Miller, Jr., Department of Obstetrics and Gynecology, Louisiana State University Medical Center, 1542 Tulane Avenue, New Orleans, LA 70112-2822.

Received 5 February 1997
Accepted 23 June 1997

oral medication was administered. Patients were questioned later as to the occurrence of side effects, including nausea, vomiting, diarrhea, abdominal pain, respiratory difficulty, or skin rash. Information was obtained by patient recall at a subsequent visit; diaries were not kept. Patients were instructed to abstain from intercourse until their sexual partners had completed treatment. Partners were referred to a sexually transmitted disease (STD) clinic available to all patients. The occurrence of this STD was reported to the State Health Department. A test of cure was generally obtained 2 weeks after treatment, a time selected to fit with general clinic activity. A specific study reappointment was not possible. Patient age, race, parity, and pregnancy outcome (gestational age and birth weight) were obtained from chart review and subsequent patient contact. The presence of positive tests for other STDs at any time during pregnancy was recorded. These included chlamydia by Gen-Probe, hepatitis B surface antigen (HBsAg), human immunodeficiency virus (HIV) antibody confirmed by Western blot, veneral disease research laboratory (VDRL) reaction, and human papilloma virus (HPV) by Papanicolaou (Pap) smear. These tests were obtained at the initial visit. VDRL, HIV, and Gen-Probe for chlamydia and gonorrhea were repeated at 34 weeks. Patients with signs or symptoms were cultured for genital herpes, but culture was not routinely obtained. Some patients were previously included in a report on use of azithromycin.[6]

### RESULTS

Treated patients were young. Maternal age ranged from 14 to 37 years, with a median of 18 years. There were 68 nulliparous patients; 15 had 1 prior delivery, 12 had 2, and 7 had ≥3. Patients were African-American except for one Caucasian. Treatment was done in 33 patients prior to 14 weeks gestation. Treatment between 14 and 27 weeks was done in 47 patients, and 22 patients were treated at 28 weeks or greater.

Other STDs were frequently identified. Chlamydial carriage was found in 59 patients. Concomitant infection was present in 38. Six patients had a reactive VDRL. Five patients had evidence of HPV on Pap smear. Three patients were HBsAg positive. Six treated patients did not have gonorrhea at the time of initial obstetric evaluation, but subsequently acquired this STD.

Cefixime was well tolerated; only two patients, both of whom also received azithromycin, experienced nausea and vomiting. A third patient receiving no other antibiotics had diarrhea. No patient reported a skin rash or respiratory difficulty. Patients received azithromycin 30 min prior to cefixime in 37 cases. Other antibiotics initiated the same day include erythromycin (1), metronidazole (3), clindamycin gel (1), ampicillin (2), cephalexin (1), and terconazole cream (1).

Treatment was successful in 79 of 83 patients who had tests of cure. Nineteen patients were either delivered (5), underwent pregnancy termination (2), transferred (2), or lost to follow-up (10). The four patients (4.8%) who failed therapy had tests of cure obtained at 20, 29, 39, and 55 days post-treatment. The patients were 19, 16, 18, and 15 years old, respectively. Reinfection, defined by a subsequent positive test after the test of cure was negative, was found in 3 of 44 (6.8%) patients who were retested during the same pregnancy. For these 3 patients maternal age was 15, 16, and 17 years. Retreatment with cefixime was done for reinfection and treatment failure. It was effective in all 7. Retreatment is not included in efficacy or side effect data.

Delivery information was sought from review of records and follow-up contact with patients, when available. One patient aborted 3 weeks after treatment at 18 weeks gestation. Delivery at <37 weeks occurred in 10 of 74 patients. Birth weight was <2,500 g in 15 of 74 patients; only 1 of these was <1,500 g.

### DISCUSSION

A single 400 mg dose of cefixime was found to be an effective treatment of *Neisseria gonorrhoeae* endocervical carriage in pregnant women. Side effects did not pose a major problem. Single dose treatment for pregnant women is not new, but this orally administered medication is easier to give. We believe there is little risk for this class B medication. Our reported cure rate in pregnant women is similar to that of ceftriaxone (95%).[7]

Our failure rate (4.8%) was quite similar to our demonstrated reinfection rate (6.8%). We suspect that many failures were actually reinfection. Both treatment failure and reinfection appeared in younger patients; all were teenagers. Programs similar to ours may need to reemphasize the importance of condom use in the prevention of STDs combined with appropriate evaluation, treatment, and follow-up of sexual partners.[1]

Both low birth weight (20.2%) and preterm delivery (13.5%) are consistent with the patient population served,[3] but are causes for concern.

We note that a majority of reported patients also had cervical carriage of chlamydia during the studied pregnancy. This may be attributed to the age (primarily adolescents) and other demographic data (poverty, inner city, race) in conjunction with an existing diagnosis of an STD.

For populations similar to ours, we believe that initial and late screening for gonorrhea is appropriate. Treatment with oral cefixime provides an effective and well-tolerated cure which can be easily administered.

### REFERENCES

1. Sexually transmitted disease guidelines. MMWR 42(RR-14):52, 1993.
2. Moran JS, Levin WC: Drugs of choice for the treatment of uncomplicated gonococcal infections. Clin Infect Dis 20:S47–65, 1995.
3. Boudreaux MC, Miller JM Jr, Wightkin J, Martin S, Mather F: Collaborative care for low and high risk obstetric patients: An evolving model. J Perinatol 17:33–36, 1997.
4. Hosein IK, Kaunitz AM, Craft SJ: Detection of cervical *Chlamydia trachomatis* and *Neisseria gonorrhoeae* with deoxyribonucleic acid probe assays in obstetric patients. Am J Obstet Gynecol 167:588–591, 1992.
5. Hale YM, Melton ME, Lewis JS, Willis DE: Evaluation of PACE 2 *Neisseria gonorrhoeae* assay by three public health laboratories. Clin Microbiol 31:451–453, 1993.
6. Miller JM Jr: Efficacy and tolerance of single dose azithromycin for the treatment of chlamydial cervicitis during pregnancy. Infect Dis Obstet Gynecol 3:189–192, 1996.
7. Carrence MR, Farris JR, Spalding TR, Barnes DL, Castaneda YS, Wendel GD Jr: Treatment of gonorrhea in pregnancy. Obstet Gynecol 81:33–38, 1993.
